# Effects of Te- and Fe-doping on the superconducting properties in Fe_*y*_Se_1−__*x*_Te_*x*_ thin films

**DOI:** 10.1038/s41598-021-04403-4

**Published:** 2022-01-10

**Authors:** Yalin Zhang, Tong Wang, Zhihe Wang, Zhongwen Xing

**Affiliations:** 1 National Laboratory of Solid State Microstructures and Collaborative Innovation Center of Advanced Microstructures, Nanjing University, Nanjing, 210093 China; 2 College of Engineering and Applied Sciences, Nanjing University, Nanjing, 210093 China; 3 Department of Mathematics, Nanjing University of Aeronautics and Astronautics, Nanjing, 210016 China; 4 School of Physics, Nanjing University, Nanjing, 210093 China; 5 School of Electronic Science and Engineering, Nanjing University, Nanjing, 210093 China

**Keywords:** Superconducting properties and materials, Surfaces, interfaces and thin films

## Abstract

High quality Fe_*y*_Se_1−*x*_Te_*x*_ epitaxial thin films have been fabricated on TiO_2_-buffered SrTiO_3_ substrates by pulsed laser deposition technology. There is a significant composition deviation between the nominal target and the thin film. Te doping can affect the Se/Te ratio and Fe content in chemical composition. The superconducting transition temperature *T*_*c*_ is closely related to the chemical composition. Fe vacancies are beneficial for the Fe_*y*_Se_1−*x*_Te_*x*_ films to exhibit the higher *T*_*c*_. A 3D phase diagram is given that the optimize range is *x* = 0.13–0.15 and *y* = 0.73–0.78 for Fe_*y*_Se_1−*x*_Te_*x*_ films. The anisotropic, effective pining energy, and critical current density for the Fe_0.72_Se_0.94_Te_0.06_, Fe_0.76_Se_0.87_Te_0.13_ and Fe_0.91_Se_0.77_Te_0.23_ films were studied in detail. The scanning transmission electron microscopy images display a regular atomic arrangement at the interfacial structure.

## Introduction

In 2008, Kamihara et al.^1^ first discovered the iron-based superconductor LaO_1−*x*_F_*x*_FeAs, which has a superconducting critical temperature of 26 K. Subsequently, Hsu et al*.*^2^ reported that the binary superconductor FeSe with antifluorite planes has the transition temperature of 8 K. Through the applied pressure on the samples, the transition temperature can reach ~ 37 K^3,4^. Ge et al.^5^ reported a superconducting transition temperature above 100 K in single-layer FeSe film grown on Nb-doped SrTiO_3_ (STO) substrate by molecular beam epitaxy method. Due to its simple crystal structure, this binary FeSe system with higher *T*_*c*_ is available, which has attracted tremendous interest in exploring the mechanism of high-temperature superconductivity^6–8^. Generally, the FeSe layer is responsible for the superconductivity and the paired electrons are mainly 3d electrons of Fe ions. Meanwhile, the FeSe layers exhibit electrical neutrality, and the atoms between the layers are bonded together by van der Waals^9,10^. However, the same structure as FeTe does not show superconducting behavior. Yeh et al.^11^ found that when Te atoms are replaced by partially substituted Se atoms, the antiferromagnetic can be suppressed and its superconductivity is induced with a superconducting transition temperature of 15 K. In bulk crystals, the optimal Te content to achieve the highest *T*_*c*_ is considered to be *x* ≈ 0.6, and phase separation occurs in the region of 0.1 ≤ *x* ≤ 0.3^12^. Liu et al.^13^ have studied the electronic and magnetic phase diagram of Fe_1.02_Se_*x*_Te_1−*x*_ single crystal superconductors. They showed that the phase diagram contains three regions, namely long-range antiferromagnetic order with a wave vector (*π*, 0) in region I (0 ≤ *x* < 0.09), neither long-range antiferromagnetic order nor bulk superconductivity in Region II (0.09 < *x* < 0.29) and the evidence of bulk superconductivity with the *T*_*c*_ about 14.5 K in Region III (*x* ≥ 0.29). The phase diagram of FeSe_1−*x*_Te_*x*_ films on CaF_2_ substrates showed that the maximum value of *T*_*c*_ is as high as 23 K at *x* = 0.2, and a sudden suppression of *T*_*c*_ is observed at 0.1 < *x* < 0.2, whereas *T*_*c*_ increases with decreasing *x* for 0.2 ≤ *x* < 1^14^. The interface effect between film and substrate makes it possible to obtain the Fe_*y*_Se_1−*x*_Te_*x*_ films with high transition temperature in a metastable phase. Although researchers have done many studies on superconducting mechanism of Fe(Se, Te) films that prepared by pulsed laser deposition (PLD), the bidirectional effect of chemical composition on the superconductivity of Fe_*y*_Se_1−*x*_Te_*x*_ films is uncertain^15–19^. In this paper, we have prepared polycrystalline targets with different nominal composition to grow Fe_*y*_Se_1−*x*_Te_*x*_ films and did a detailed investigation on the superconducting properties and its phase diagram. The experimental results show that there is a significant deviation between the nominal composition of targets and the real composition of films. The increase of Te doping can have an impact not only on Se/Te ratio but also Fe content. The electrical transport results indicate that the optimal range of Te and Fe content is *x* = 0.13–0.15 and *y* = 0.73–0.78 for Fe_*y*_Se_1−*x*_Te_*x*_ films with excellent superconductivity. As *x* = 0.13, *y* = 0.76, the maximum of zero-resistivity temperature *T*_*c*_^*0*^_*max*_ of film is over 17 K and the critical current density *J*_*c*_ is higher than 10^6^ A/cm^2^ at 4 K. Moreover, STEM images reveal that the interface region of Fe_*y*_Se_1−*x*_Te_*x*_/TiO_2_/SrTiO_3_ heterostructure is sharp and clean, and no obvious atomic diffusion and migration are detected.

## Results and discussion

In the published papers^14,20–23^, authors usually defined the nominal composition of the targets as the real composition of Fe_*y*_Se_1−*x*_Te_*x*_ films. However, the deviation between the nominal composition and the real composition may affect the study on the mechanism of superconductivity for Fe_*y*_Se_1−*x*_Te_*x*_ films. We determined the real composition of films by EDX mapping in SEM technology. Our experimental results show that there is a significant deviation between the nominal composition and the real composition in two groups, as shown in Tables 1 and 2. At the first, we fixed the content of Fe and adjusted the amount of Te doping in targets (nominal composition in Table 1). EDX results show that Te doping can have an impact not only on Se/Te ratio but also the Fe content in films. The optimal chemical composition may play an important role in films with the excellent superconducting property. Base on this result, we measured the superconducting properties of these films and gave them in the following text. To explore the effect of Fe content on the superconductivity of Fe_*y*_Se_1−*x*_Te_*x*_ films, we fixed the Se/Te ratio and change the Fe doping in the nominal composition, as shown in Table 2. It can be seen that the change of Fe doping in the nominal composition also affects the Fe content in the real composition, but has little influence on the ratio of Se/Te. During the deposition, the transfer and growth rate of Fe/Se/Te elements are different, which may result in the obvious deviation of chemical composition between target and film. Therefore, we think that it is inaccurate to directly define the nominal composition of the targets as the real composition of the films.

**Table 1:** The composition, onset and zero-resistivity temperature, and *c*-axis lattice parameter of thin films for nominal composition FeSe_1−*x*_Te_*x*_ targets.

Nominal composition	Real composition (± 0.02)	*T*_*c*_^*onset*^ (K)	*T*_*c*_^*0*^ (K)	*c* parameter (Å)
FeSe_0.6_Te_0.4_	Fe_0.63_Se_0.97_Te_0.03_	5.49	3.71	5.6361
FeSe_0.5_Te_0.5_	Fe_0.72_Se_0.94_Te_0.06_	10.73	9.44	5.7526
FeSe_0.4_Te_0.6_	Fe_0.76_Se_0.87_Te_0.13_	18.95	17.34	5.8398
FeSe_0.3_Te_0.7_	Fe_0.91_Se_0.77_Te_0.23_	16.13	14.35	5.9486
FeSe_0.2_Te_0.8_	Fe_1.09_Se_0.66_Te_0.34_	13.21	11.37	6.0502
FeSe_0.1_Te_0.9_	Fe_1.43_Se_0.44_Te_0.56_	8.03	–	6.1973

**Table 2:** The composition, onset and zero-resistivity temperature, and *c*-axis lattice parameter of thin films for nominal composition Fe_*y*_Se_0.4_Te_0.6_ targets.

Nominal composition	Real composition (± 0.02)	*T*_*c*_^*onset*^ (K)	*T*_*c*_^*0*^ (K)	*c* parameter (Å)
Fe_0.9_Se_0.4_Te_0.6_	Fe_0.73_Se_0.85_Te_0.15_	20.35	17.55	5.7287
FeSe_0.4_Te_0.6_	Fe_0.76_Se_0.87_Te_0.13_	18.95	17.34	5.8398
Fe_1.1_Se_0.4_Te_0.6_	Fe_0.78_Se_0.84_Te_0.16_	17.64	16.01	6.0047

**Figure 1:**
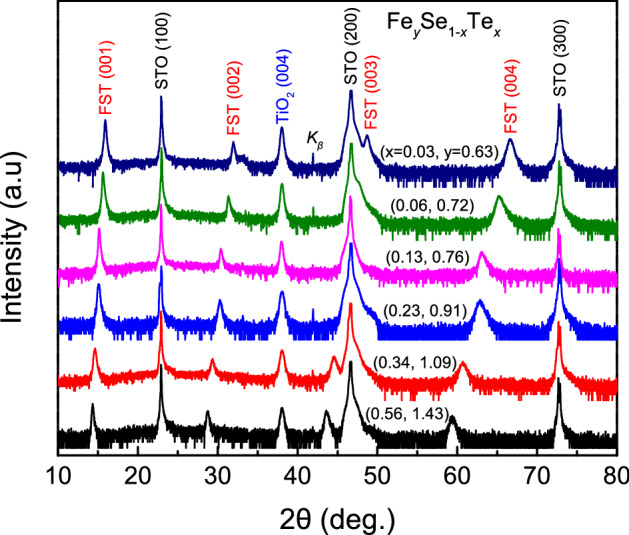
Semilogarithmic X-ray diffraction patterns of Fe_*y*_Se_1−*x*_Te_*x*_ thin films.

The semilogarithmic XRD patterns of Fe_*y*_Se_1−*x*_Te_*x*_ films are shown in Fig. 1. From Fig. 1, only Fe_*y*_Se_1−*x*_Te_*x*_ and TiO_2_ peaks are observed along the *c*-axis (00*l*), which indicates the Fe_*y*_Se_1−*x*_Te_*x*_ films to be the single tetragonal phase. Our previous work confirmed that TiO_2_ as a buffer layer could increase the lattice match between Fe(Se, Te) film and STO substrate, so as to enhance the superconducting property of Fe(Se, Te) film^24^. We find that with increasing Te doping, the (00*l*) peaks significantly shift to a low angle. The *c*-axis lattice parameters for Fe_*y*_Se_1−*x*_Te_*x*_ films are obtained by fitting the (001) peak, as listed in Table 1. The ionic radius of Te (Te^2−^, 221 pm) is larger than that of Se (Se^2−^, 198 pm)^25^. Te doping can increase the distance between the Fe plane and Se/Te atom (*h*_*Fe-Se/Te*_), which increases the *c*-axis lattice parameters. Zhuang et al*.*^26^ and Imai et al*.*^27^ have reported the effect of chemical composition on the structure in FeSe_1−*x*_Te_*x*_ films. In our results, the increase of Te doping in targets can also raise the Fe content in Fe_*y*_Se_1−*x*_Te_*x*_ films. For *y* > 1 in Table 1, we think that the additional Fe may be incorporated in the inter-layer of Fe-Se/Te space. Thus, Fe content plays a part in the change of lattice parameter*.* Zhuang et al*.*^22^ assumed that two key factors affected the lattice parameters of thin films under the Fe-deficient conditions. The ionic radius of Fe is smaller than that of Se and Te. Fe vacancy phase leads to a smaller *c*-axis lattice parameter, while Se/Te interstitial phase leads to a larger *c*-axis in comparison with the stoichiometric phase. For Table 2, with increasing the Fe doping, the *c*-axis lattice parameter of films increases. The above results show that the superconducting structures of Fe_*y*_Se_1−*x*_Te_*x*_ films are not changed with 0.63 < *y* < 1.43, whereas Te and Fe doping jointly influence the *c*-axis lattice parameter.

**Figure 2:**
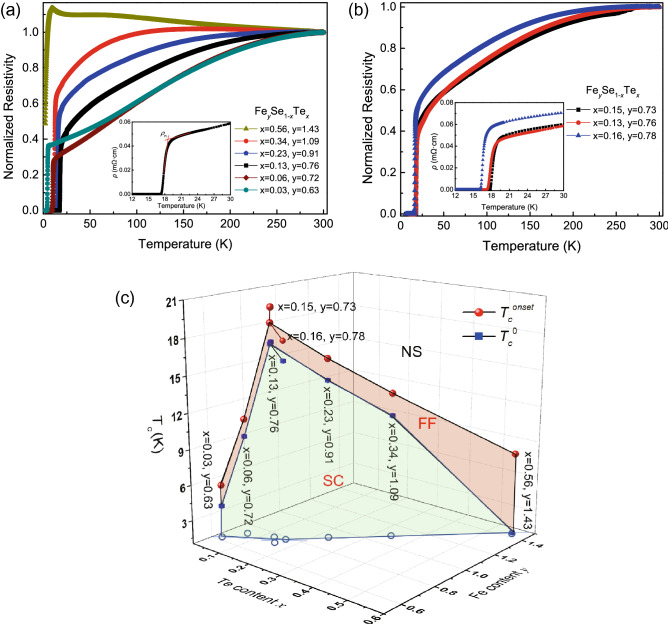
(**a**,**b**) Temperature dependence of resistivity from 2 to 300 K for Fe_*y*_Se_1−*x*_Te_*x*_ thin films. (**a**) (*x*, *y*) = (0.03, 0.63), (0.06, 0.72), (0.13, 0.76), (0.23, 0.91), (0.34, 1.09) and (0.56, 1.43). Inset: enlarged plot for the definition of normal-state resistivity *ρ*_n_. (**b**) (*x*, *y*) = (0.15, 0.73), (0.13, 0.76) and (0.16, 0.78). Inset: the enlarged *ρ*-*T* curve near *T*_*c*_. (**c**) Sketch of the proposed temperature doping 3D phase diagram for Fe_*y*_Se_1−*x*_Te_*x*_ superconducting system, showing regions of superconductivity (SC), flux flow (FF) and normal state (NS).

Figure 2a shows the temperature dependence of the normalized resistivity *ρ/ρ*_300K_ (*ρ*-T) for the Fe_*y*_Se_1−*x*_Te_*x*_ films. For 0.03 ≤ *x* ≤ 0.23 in Fig. 2a, as the temperature above the superconducting transition, the films only display metallic behavior. However, for *x* > 0.23, the resistivity of films changes from semiconducting to metallic before superconducting transition. This change may attribute to the structural phase transition and magnetic phase transition caused by Te doping. If we define the point of intersection of the two lines as the normal-state resistivity *ρ*_n_, as shown in the inset of Fig. 2a, the onset transition temperature *T*_*c*_^*onset*^ and zero-resistivity temperature *T*_*c*_^*0*^ are obtained from these *ρ-T* curves where the resistivity is 90% and 1% of the normal state resistivity *ρ*_n_, respectively. The values of *T*_*c*_^*onset*^ and *T*_*c*_^*0*^ for these films are listed in Table 1 and plotted in the 3D phase diagram, as shown in Fig. 2c. With increasing the Te doping, the *T*_*c*_ rises at first and then decreases. From Fig. 2c, the Fe_0.76_Se_0.87_Te_0.13_ film exhibits the higher *T*_*c*_^*onset*^ and *T*_*c*_^*0*^ about 18.95 K and 17.34 K, respectively. Surprising us, the composition of the Fe_0.76_Se_0.87_Te_0.13_ film is not consistent with that of the single crystal, where the highest *T*_*c*_ is considered *x* ≈ 0.6 in Fe(Se_1−*x*_Te_*x*_)_0.82_ polycrystal sample, and located at the phase separation region of 0.1 ≤ *x* ≤ 0.3^12^. They argued that the single-phase of Fe(Se_1−*x*_Te_*x*_)_0.82_ single crystals with the region of 0.1 ≤ *x* ≤ 0.3 were not easy to obtain. However, Imai et al.^14^ assumed that the single-phase epitaxial films of FeSe_1−*x*_Te_*x*_ with 0.1 ≤ *x* ≤ 0.4 could be successfully prepared on CaF_2_ substrates, attributing to the strain effect between film and substrate. Due to the different substrates, there is a difference in the suppression of phase separation and giant enhancement of *T*_*c*_ for Fe_*y*_Se_1−*x*_Te_*x*_ films. Our experimental results display that the sudden suppression of *T*_*c*_ is observed at 0.03 ≤ *x* < 0.13, whereas *T*_*c*_ increases with decreasing *x* for 0.13 ≤ *x* < 0.56. The superconductivity is related to the Te and Fe content in Fe_*y*_Se_1−*x*_Te_*x*_ films. Therefore, we must consider the effects of Fe vacancies on the superconductivity of Fe_*y*_Se_1−*x*_Te_*x*_ films. Figure 2b shows the temperature dependence of the normalized resistivity *ρ/ρ*_300K_ (*ρ*-T) near the optimal composition Fe_*y*_Se_1−*x*_Te_*x*_ films, where *x* ~ 0.15 and *y* ~ 0.76. The results demonstrate the effects of Fe vacancies on the superconductivity of Fe_*y*_Se_1−*x*_Te_*x*_ films. The *T*_*c*_^*onset*^ and *T*_*c*_^*0*^ are listed in Table 2 and plotted in the 3D phase diagram of Fig. 2b. Although we do not know why the *T*_*c*_^*onset*^ and *T*_*c*_^*0*^ increase with decreasing the Fe content near *y* = 0.76, the transition width broadens much more. This result further confirms that the optimal range is *x* = 0.13–0.15 and *y* = 0.73–0.78 for the Fe_*y*_Se_1−*x*_Te_*x*_ films.

Figure 2c is a new 3D phase diagram for the Fe_*y*_Se_1−*x*_Te_*x*_ films. The blue open symbols are the projection of experimental points on the *xy*-plane at *T*_*c*_ ≈ 1 K. The 3D phase diagram can be divided into three regions by *T*_*c*_^*onset*^(*x*, *y*) and *T*_*c*_^*0*^(*x*, *y*) curved surfaces, which are superconductivity (SC), flux flow (FF), and normal state (NS), respectively. Above the *T*_*c*_^*onset*^(*x*, *y*) curved surfaces, the Fe_*y*_Se_1−*x*_Te_*x*_ film is in the normal state. Below the *T*_*c*_^*0*^(*x*, *y*) curved surfaces, the Fe_*y*_Se_1−*x*_Te_*x*_ film is in a superconducting state. Between the *T*_*c*_^*onset*^(*x*, *y*) and *T*_*c*_^*0*^(*x*, *y*) curved surfaces, the Fe_*y*_Se_1−*x*_Te_*x*_ film is in the flux flow state. The 3D phase diagram demonstrates that the phase separation is absent, and that the optimal composition for the Fe_*y*_Se_1−*x*_Te_*x*_ film on STO substrate is not *x* ≈ 0.5 and *y* = 1 but *x* ~ 0.13 and *y* ~ 0.76. It should be noted that the dependence of *T*_*c*_ on *x* suddenly changes at the boundary defined by 0.03 ≤ *x* < 0.13 in our experiment. Thus, not only the decrease of *T*_*c*_ with *x* ≥ 0.13 can be explained by the empirical law that shows the relation between *T*_*c*_ and structural parameters, but also the sudden suppression of *T*_*c*_ in films with 0.03 ≤ *x* < 0.13 can be explained by the orthorhombic distortion results in a suppression of *T*_*c*_. As reported by Imai et al*.*^14^, the orthorhombic distortion is applicable to the behavior of films, if a large orthorhombic distortion is observed only in films with 0 < *x* < 0.1, which is consistent with our result of 0.03 ≤ *x* < 0.13. Chen et al*.*^28^ and Bendele et al*.*^29^ pointed out that a few Fe vacancies were beneficial to improve the superconductivity and raised the superconducting transition temperature for Fe_*y*_Se_1−*x*_Te_*x*_ films. The inhomogeneous distribution of Fe vacancies can induce the Fe disorder effect in Fe_*y*_Se_1−*x*_Te_*x*_ films with *y* < 1. The first-principles calculation also showed that the Fe vacancies could effectively increase the number of electron carriers and change the electronic properties in samples^22^. Therefore, in this experiment, the highest *T*_*c*_^*onset*^ and *T*_*c*_^*0*^ occur near *y* = 0.76. When the Te and Fe content exceed the optimal composition, the *T*_*c*_^*onset*^ and the *T*_*c*_^*0*^ of Fe_*y*_Se_1−*x*_Te_*x*_ films decrease. For example, as *x* = 0.56, *y* = 1.43, the *ρ* does not down to 1% *ρ*_n_, so the Fe_1.43_Se_0.44_Te_0.56_ film only has the *T*_*c*_^*onset*^ about 8.03 K.

**Figure 3:**
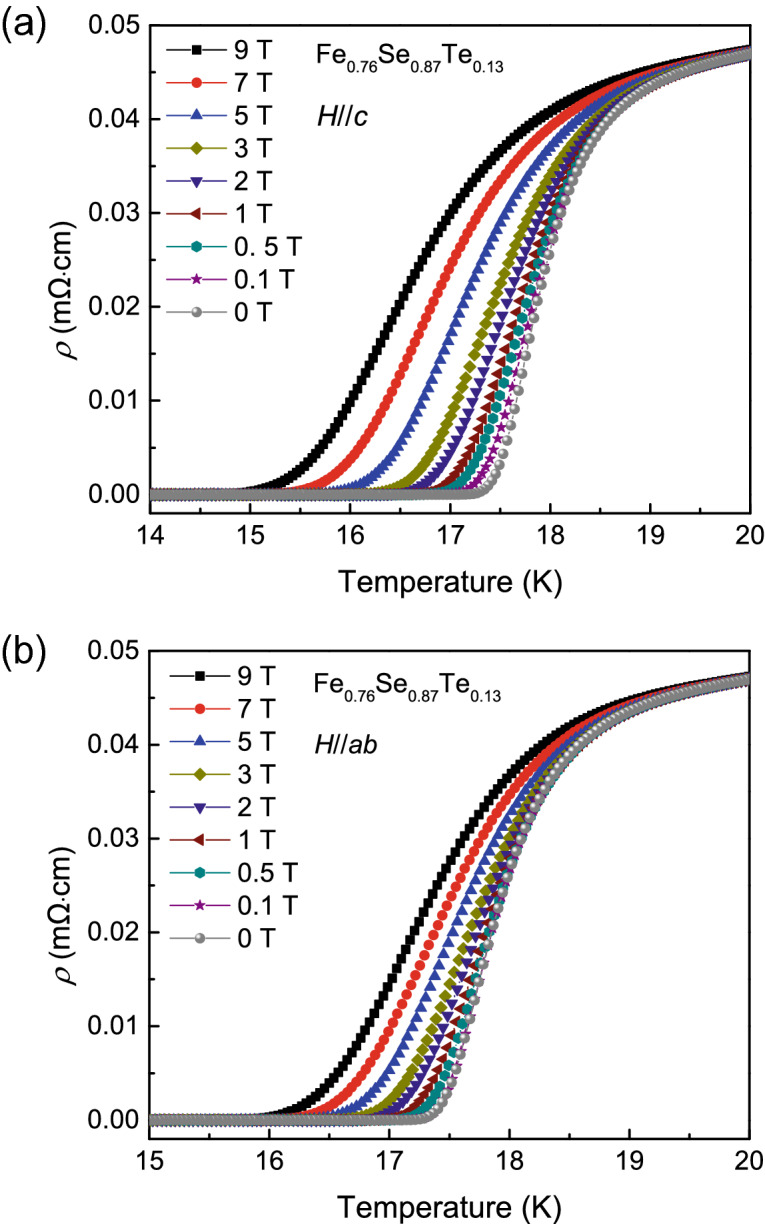
Temperature dependence of resistivity near *T*_*c*_ in various magnetic fields for Fe_0.76_Se_0.87_Te_0.13_ thin film. (**a**) *H*//*c* and (**b**) *H*//*ab*.

To understand the new phase diagram, we have measured the electrical transport and magnetization properties for Fe_*y*_Se_1−*x*_Te_*x*_ films in magnetic field. Here, we choose some typical results in the next part. Figure 3a,b present the temperature dependence of resistivity of Fe_0.76_Se_0.87_Te_0.13_ film in various magnetic fields up to 9 T applied perpendicular and parallel to the *c*-axis. With increasing the applied magnetic field, the resistive transition is broadened. At the same field, the width of superconducting transition Δ*T*_*c*_ for *H*//*c* is larger than that for *H*//*ab*. This result indicates that the Fe_*y*_Se_1−*x*_Te_*x*_ films are anisotropic near *T*_*c*_.

**Figure 4:**
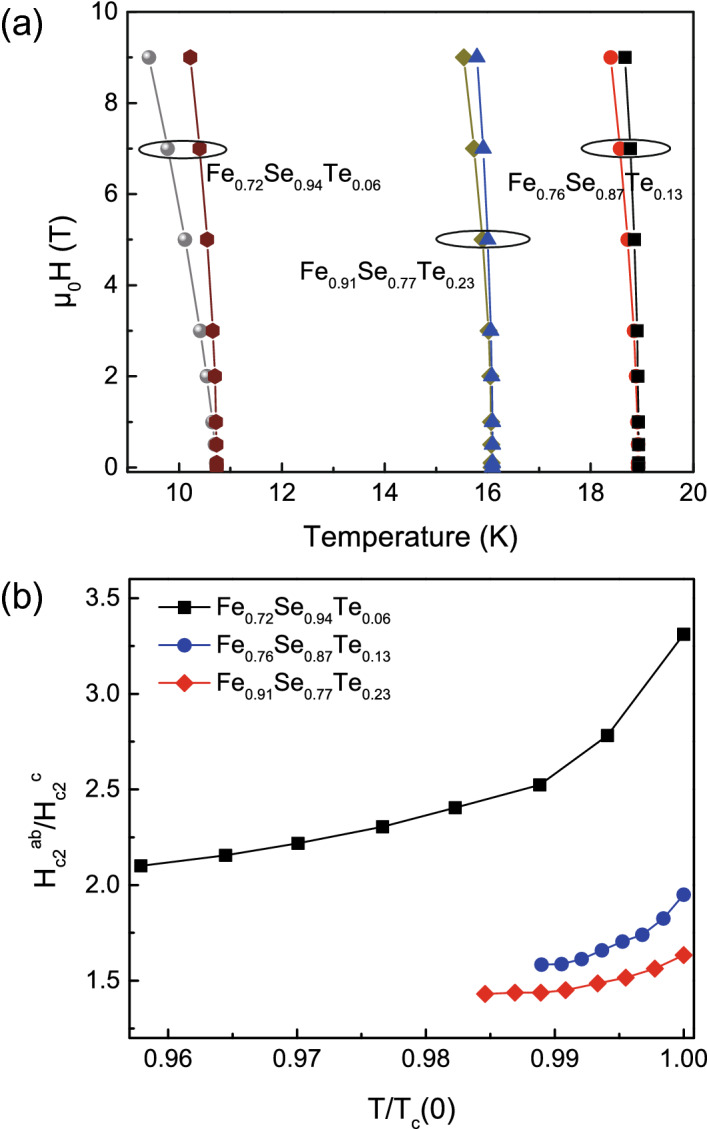
(**a**) Upper critical field versus temperature phase diagram determined by *ρ*/*ρ*_*n*_ = 90%. (**b**) Temperature dependence of anisotropy for Fe_0.72_Se_0.94_Te_0.06_, Fe_0.76_Se_0.87_Te_0.13_ and Fe_0.91_Se_0.77_Te_0.23_ thin films.

If we define the onset transition temperature *T*_*c*_^*onset*^ as the critical temperature *T*_*c*_, namely the field is the upper critical field *H*_*c2*_, we can get the temperature of the upper critical field near *T*_*c*_. The *H*-*T* phase diagram for Fe_0.72_Se_0.94_Te_0.06_, Fe_0.76_Se_0.87_Te_0.13_ and Fe_0.91_Se_0.77_Te_0.23_ films is shown in Fig. 4a. The temperature dependence of the upper critical field *H*_*c*2_ near *T*_*c*_ follows the formula $H_{c2} (T) = H_{c2} (0)(1 - T/T_{c} )^{n}$, where *H*_*c*2_ (0) and *n* are parameters obtained from the experimental data. The parameters *H*_*c*2_(0) and *n* near *T*_*c*_ for (1) Fe_0.72_Se_0.94_Te_0.06_, (2) Fe_0.76_Se_0.87_Te_0.13_ and (3) Fe_0.91_Se_0.77_Te_0.23_ films, respectively, are (1) 67.9 T and 0.63 for *H*//*ab*, 46.6 T and 0.78 for *H*//*c*; (2) 91.8 T and 0.54 for *H*//*ab*, 82.5 T and 0.57 for *H*//*c*; and (3) 77.4 T and 0.71 for *H*//*ab*, 60.2 T and 0.53 for *H*//*c*. The result implies that the upper critical field *H*_*c*2_ depends on Te and Fe content. The higher upper critical field *H*_*c2*_ located at *x* = 0.13–0.15 and *y* = 0.73–0.78 for Fe_*y*_Se_1−*x*_Te_*x*_ films. From Fig. 4a, we can get the temperature dependence of the anisotropic factor *γ* = *H*_*c*2_^*ab*^/*H*_*c*2_^*c*^ near *T*_*c*_, as shown in Fig. 4b. We can see that the *γ* value decreases with decreasing temperature. $\gamma_{{T_{c} }}$ for Fe_0.72_Se_0.94_Te_0.06_, Fe_0.76_Se_0.87_Te_0.13_ and Fe_0.91_Se_0.77_Te_0.23_ films are estimated about 3.3, 1.9 and 1.6, respectively. Increasing Te doping can inhibit its anisotropy and enhance the correlation between Fe-Se/Te layers, leading to the increasing the dimensionality of Fermi surface, which is conducive to the transmission of electrons along the *c*-axis direction.

**Figure 5:**
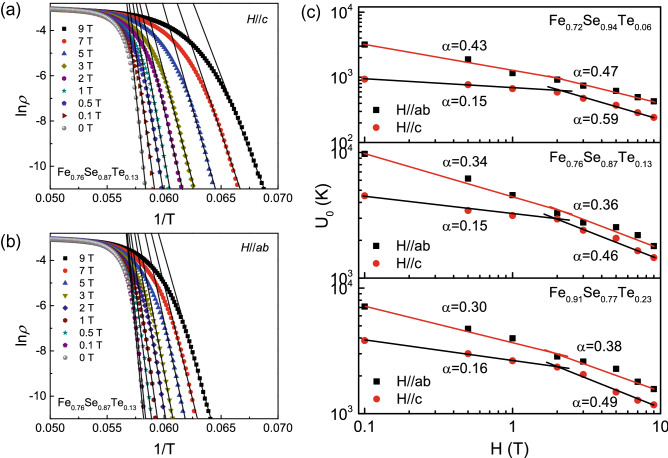
ln*ρ* versus 1/T curves in various magnetic fields of Fe_0.76_Se_0.87_Te_0.13_ thin film. (**a**) *H*//*c*; (**b**) *H*//*ab*. (**c**) Magnetic field dependence of the effective flux pinning energy for Fe_0.72_Se_0.94_Te_0.06_, Fe_0.76_Se_0.87_Te_0.13_ and Fe_0.91_Se_0.77_Te_0.23_ thin films.

The effective pining energy is an important parameter to enhance the capacity of carrying current for superconducting materials. According to the thermally activated flux flow (TAFF) theory, the ln*ρ*–1/T in the TAFF region can be described using an Arrhenius relation^30^, $\rho = \rho_{0} \exp \left( { - \frac{{U_{0} }}{{{\rm K}_{B} {\rm T}}}} \right)$ where *U*_*0*_ is the effective pinning energy. Figure 5a,b shows the linear relationship between ln*ρ* and 1/T of the Fe_0.76_Se_0.87_Te_0.13_ film. From the absolute slope of ln*ρ* − 1/T curves, we can obtain the effective pinning energy *U*_*0*_ of Fe_0.72_Se_0.94_Te_0.06_, Fe_0.76_Se_0.87_Te_0.13_ and Fe_0.91_Se_0.77_Te_0.23_ films, respectively, as shown in Fig. 5c. It can be found that the *U*_*0*_ value of Fe_0.76_Se_0.87_Te_0.13_ is larger than that of Fe_0.72_Se_0.94_Te_0.06_ and Fe_0.91_Se_0.77_Te_0.23_ in the same field. What’s more, *U*_*0*_ values for *H*//*ab* plane are much higher than that for *H*//*c*, indicating the flux pinning is anisotropic. The magnetic field dependence of *U*_*0*_ follows a power low *U*_*0*_ (H) ~ *H*^-*α*^. When *H*//*ab*, the parameter *α* decreases with increasing the Te doping. The parameter *α* for the Fe_0.76_Se_0.87_Te_0.13_ and Fe_0.91_Se_0.77_Te_0.23_ films is close. For *H*//*c*, there is an obvious crossover occurred at *H* ≈ 2 T. Below 2 T, the parameter *α* is close to 0.15. Above 2 T, *α* is close to 0.5. Similar behavior has been observed in other superconductors^30–34^. In the field below 2 T, the pinning energy *U*_*0*_ is weakly dependent on the applied magnetic field *H*. It can be considered that the number of magnetic flux lines is much less than the number of pinning centers. The single vortex pinning dominates in this region^35^. As the magnetic field increases above 2 T, more flux lines enter the superconductor and the flux spacing becomes smaller, which leads to the pinning energy being inhibited. The pinning energy *U*_*0*_ becomes strongly dependent on the field *H*. and the collective creep pinning is dominant in this region^36,37^.

**Figure 6:**
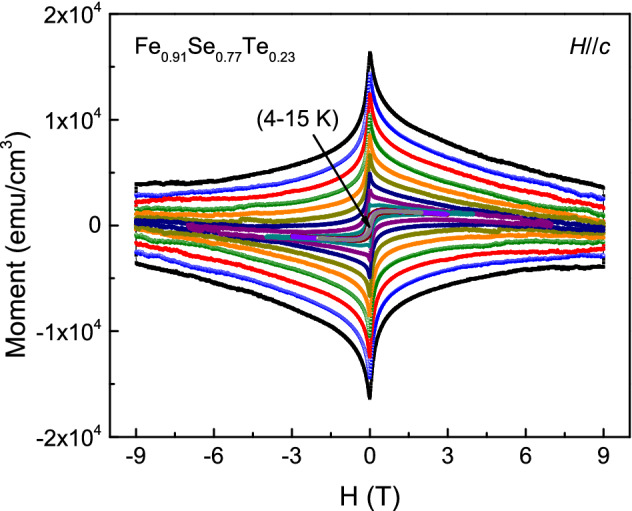
Magnetic hysteresis loops of Fe_0.91_Se_0.77_Te_0.23_ thin film at various temperatures in magnetic field parallel to the *c*-axis.

**Figure 7:**
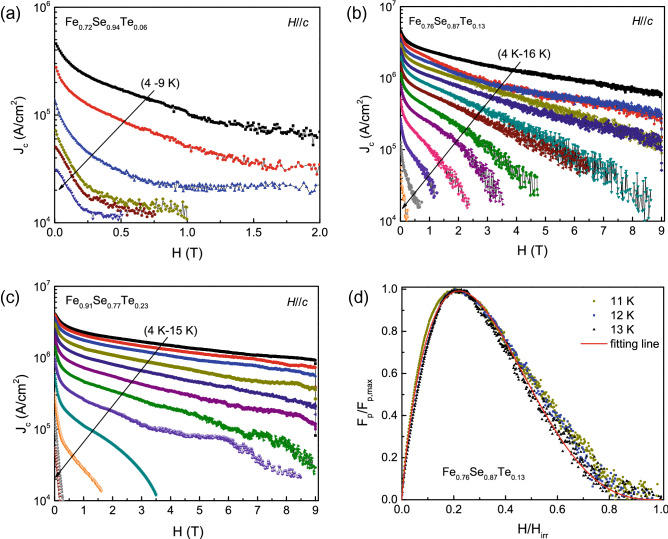
Magnetic field dependence of critical current density *J*_*c*_ at various temperatures for (**a**) Fe_0.72_Se_0.94_Te_0.06_, (**b**) Fe_0.76_Se_0.87_Te_0.13_, and (**c**) Fe_0.91_Se_0.77_Te_0.23_ thin films. (**d**) Normalized flux pinning force versus reduced magnetic field at 11, 12 and 13 K for Fe_0.76_Se_0.87_Te_0.13_ thin film. Solid line is the fitting curve using the Dew-Hughes model.

The critical current density *J*_*c*_ is also an important parameter for high quality epitaxial superconducting films. To study the effect of chemical composition on the critical current density of Fe_*y*_Se_1−*x*_Te_*x*_ films, we have measured the magnetization hysteresis loops in fields parallel to the *c*-axis from 0 to ± 9 T. Figure 6 shows the *M*-*H* loops of Fe_0.91_Se_0.77_Te_0.23_ film at various temperatures. The *M*-*H* loops show symmetric field dependence. As the field increases, the magnetization of film decreases. The critical current density *J*_*c*_ is estimated from the *M*-*H* loops by the Bean critical state model^38^: $J_{c} = 20\frac{\Delta M}{{a(1 - a/3b)}}$. Where △*M* = *M*(+) − *M*(−), *M*( +) and *M*(−) are the magnetizations when sweeping fields up and down, respectively. *a* and *b* (*a* < *b*) are the Fe_*y*_Se_1−*x*_Te_*x*_ film’s cross-sectional dimension. The field dependence of the critical current density *J*_*c*_ at various temperatures is shown in Fig. 7. From Fig. 7a–c, we can see that with increasing the Te doping, the field dependence of the critical current density *J*_*c*_ improves at higher field region. In addition, the measured critical temperature *T*_*c*_ in Fe_0.76_Se_0.87_Te_0.13_ is higher than that in Fe_0.91_Se_0.77_Te_0.23_. The calculated *J*_*c*_ at 4 K and 0 T for Fe_0.72_Se_0.94_Te_0.06_, Fe_0.76_Se_0.87_Te_0.13_, Fe_0.91_Se_0.77_Te_0.23_ films are about 4.46 × 10^5^ A/cm^2^, 4.51 × 10^6^ A/cm^2^ and 4.05 × 10^6^ A/cm^2^, respectively. This result displays that the higher *T*_*c*_ also contributes to improving the magnetic field dependence of *J*_*c*_ at 4 K. Therefore, the optimal composition is beneficial for Fe_*y*_Se_1−*x*_Te_*x*_ films exhibiting excellent superconductivity in lower field region.

Flux pinning force can provide a very efficient route to descript the vortex dynamics in superconductors^39,40^. Furthermore, we calculated the field dependence of the flux pinning force *F*_*p*_ = *μ*_0_*H* × *J*_c_ for temperatures at 11, 12 and 13 K, respectively. Based on the theory of Dew-Hughes^41^, the field dependence of the normalized vortex pinning force *f*_*p*_ should follow the expression $f_{p} = Ah^{p} (1 - h)^{q}$, where *h* = *H*/*H*_*irr*_, *p* and *q* are parameters that depend on the pinning centers. Figure 7d gives the relationship of normalized vortex pinning force *f*_*p*_ and reduced magnetic field *h* for Fe_0.76_Se_0.87_Te_0.13_ film. By fitting the *f*_*p*_-*h* curves, we obtain *p* = 0.67, *q* = 2.45, and *h*_*max*_ = 0.21, indicating that the flux pinning centers in film may be dominant by the core normal surface pinning (*p* = 0.5, *q* = 2, and *h*_*max*_ = 0.2)^42^.

**Figure 8:**
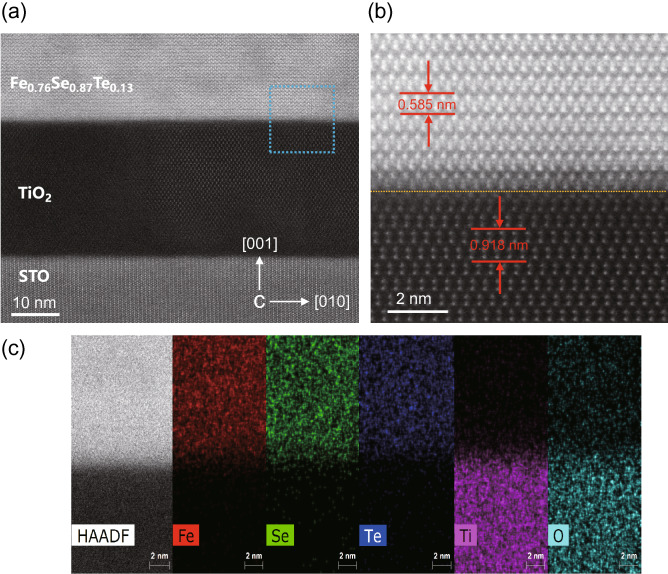
(**a**) Overview image of the Fe_0.76_Se_0.87_Te_0.13_/TiO_2_/STO thin film interface. (**b**) Atomically resolved HADDF-STEM image of Fe_0.76_Se_0.87_Te_0.13_/TiO_2_ heterostructure. (**c**) EDX-mapping results shows the distribution of Fe (red), Se (green), Te (blue) Ti (magenta), O (cyan).

The interface structure plays a vital role in determining the superconducting properties for Fe_*y*_Se_1−*x*_Te_*x*_ films. Using the STEM analysis, we could reveal the Fe_0.76_Se_0.87_Te_0.13_/TiO_2_/STO microstructure and determine the morphology of the interface. The thicknesses of Fe_0.76_Se_0.87_Te_0.13_ and TiO_2_ film are about 32.4 nm and 29.5 nm, respectively. Figure 8a shows the overview image of the Fe_0.76_Se_0.87_Te_0.13_/TiO_2_/STO interface. It can be seen that the heterostructure interface is sharp and clean. The TiO_2_ buffer was successfully deposited between the Fe_0.76_Se_0.87_Te_0.13_ film and STO substrate. Figure 8b shows the high-magnification HAADF image of Fe_0.76_Se_0.87_Te_0.13_/TiO_2_. The Fe, Se, Te, Ti and O atoms are arranged neatly at the interface. In this case, the Fe_0.76_Se_0.87_Te_0.13_ structure with a tetragonal space group P4/nmm is very simple, and each unit cell contains 3 quintuple layers (QLs), which are bonded by van der Waals (vdW)^9^. The TiO_2_ unit cell has two Ti–O triple layers, which grow on STO along the (00*l*) direction. From Fig. 8b, a nanoscale damaged layer (or transition layer) was formed between the TiO_2_ and Fe_0.76_Se_0.87_Te_0.13_ interface. To determine the formation of this transition layer, the Atomic resolution EDX mapping was conducted in this area. The chemical elemental maps of Fig. 8c confirm the suggestion from HAADF image that the atoms are arranged regularly without obvious diffusion and migration. Such high quality heterostructure has a significant influence on the enhancement of superconductivity for Fe_*y*_Se_1−*x*_Te_*x*_ films.

## Conclusion

In summary, we successfully prepared the Fe_*y*_Se_1−*x*_Te_*x*_ thin films with 0.03 ≤ *x* ≤ 0.56 and 0.63 ≤ *y* ≤ 1.43 by PLD. Our experimental results confirm the significant deviation between the nominal compositions of targets and the real compositions of Fe_*y*_Se_1−*x*_Te_*x*_ films. Chemical composition does affect the superconducting properties such as the superconducting transition temperature and the critical current density in Fe_*y*_Se_1−*x*_Te_*x*_ films. A new 3D phase diagram is presented from the experimental results of electrical transport, which reveals that the optimal composition for Fe_*y*_Se_1−*x*_Te_*x*_ films is *x* = 0.13–0.15 and *y* = 0.73–0.78. The field dependence of flux pinning energy displays that the increase of Te doping can enhance the flux pinning in Fe_*y*_Se_1−*x*_Te_*x*_ films. STEM investigation shows that the Fe_0.76_Se_0.87_Te_0.13_/TiO_2_/STO heterostructure has a sharp interface and exhibits almost no atomics intermixing. Our study results provide some further understanding on the mechanism of superconducting properties for Fe_*y*_Se_1−*x*_Te_*x*_ films, which has a certain guiding significance and reference value for the potential application of iron-based superconductors.

## Methods

The PLD targets were prepared by the self-flux method with high purity materials (Fe 99.99%, Te 99.999% and Se 99.999%) in the stoichiometric proportion. Fe, Se and Te were fully ground and squeezed into a 3/4 in. block, and then encapsulated in a vacuum quartz tube. The vacuum quartz tube was calcined in a muffle furnace at 850 °C for 72 h, then slowly cooled down to room temperature at the rate of 3 °C/min. The Fe_*y*_Se_1−*x*_Te_*x*_ epitaxial films were deposited on STO single crystalline substrates at 300 °C by PLD in a high vacuum (~ 10^–7^ mbar). The distance between target and substrate was set at ~ 70 mm. A KrF excimer laser (248 nm) was used for deposition with an energy density of 2.0 J/cm^2^ and a repetition frequency of 2 Hz. The size of the STO substrate is 5 mm × 5 mm. TiO_2_ film as a buffer layer was firstly deposited on STO substrate by PLD to improve the lattice matching between Fe_*y*_Se_1−*x*_Te_*x*_ film and STO substrate. The deposition temperature and deposition time for Fe_*y*_Se_1−*x*_Te_*x*_ and TiO_2_ films were 300 °C and 15 min, 600 °C and 4.5 min, respectively. After deposition, the films were annealed to room temperature at the rate of 5 °C/min.

X-ray diffraction (XRD) patterns using the *θ*/2*θ* method were measured by Bruker D8 with CuK*α* radiation (λ = 1.54 Å). The *Φ*-scan of (101) peak from the Fe_0.76_Se_0.87_Te_0.13_ thin film is shown in Supplementary SFig. 1. The chemical composition of Fe_*y*_Se_1−*x*_Te_*x*_ films was determined by energy dispersive *x*-ray spectroscopy (EDX) in a Gemini 500 scanning electron microscope (SEM) mapping. The measurements of electrical transport were carried out via the physical property measurement system (PPMS-9T, Quantum Design). Magnetization measurements on films with 100 Oe/s of sweep rate were performed in vibrating sample magnetometer (VSM). The microstructures of Fe_*y*_Se_1−*x*_Te_*x*_ films were examined by scanning transmission electron microscopy (STEM, FEI Titan G2 60–300 aberration). Samples for the STEM were cut and milled in a focused ion beam (FIB, FEI Helios Nanolab 600) according to the so-called micro-bridge sampling technique.

## Supplementary Information

Supplementary Figure 1.
