# Supplementary figures and images for: Effects of Te- and Fe-doping on the superconducting properties in FeySe1−xTex thin films
Source: Sci Rep. 2022 Jan 10;12:391. doi: 10.1038/s41598-021-04403-4 (PMC8748920; doi:10.1038/s41598-021-04403-4)

**Supporting**

**Fig. 1.** *Φ*-scan of (101) peak from the Fe0.76Se0.87Te0.13 thin film.


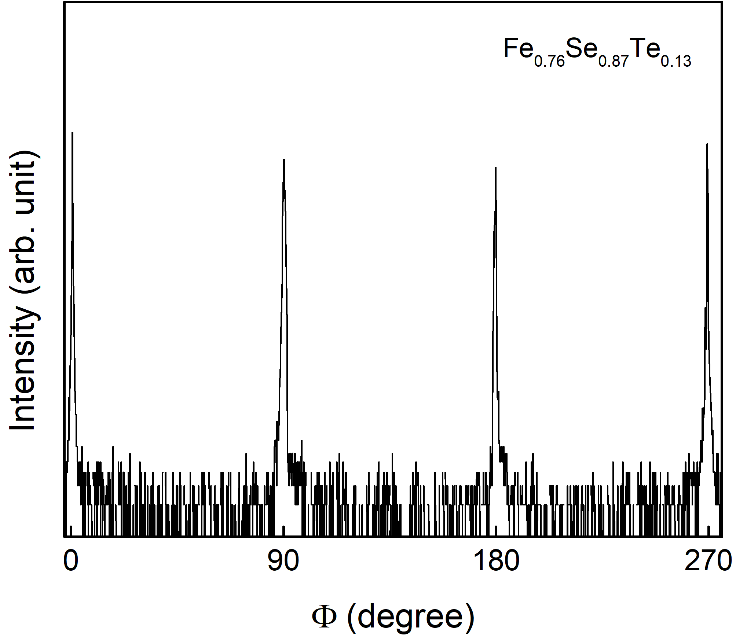

Supplement: Supplementary file 1 — Supplementary Figure 1. [file 41598_2021_4403_MOESM1_ESM.docx]
